# Gene-Wide Characterization of Common Quantitative Trait Loci for *ABCB1* mRNA Expression in Normal Liver Tissues in the Chinese Population

**DOI:** 10.1371/journal.pone.0046295

**Published:** 2012-09-26

**Authors:** Weihua Shou, Dazhi Wang, Kaiyue Zhang, Beilan Wang, Zhimin Wang, Jinxiu Shi, Wei Huang

**Affiliations:** 1 Ruijin Hospital, School of Medicine, Shanghai Jiaotong University, Shanghai, China; 2 Shanghai-MOST Key Laboratory of Health and Disease Genomics, Department of Genetics, Chinese National Human Genome Center, Shanghai, China; Central China Normal University, China

## Abstract

In order to comprehensively screen genetic variants leading to differential expression of the important human *ABCB1* gene in the primary drug-metabolizing organ, *ABCB1* mRNA expression levels were measured in 73 normal liver tissue samples from Chinese subjects. A set of Tag SNPs. were genotyped. In addition, imputation was performed within a 500 kb region around the *ABCB1* gene using the reference panels of 1,000 Genome project and HapMap III. Bayesian regression was used to assess the strength of associations by compute Bayes Factors for imputed SNPs. Through imputation and linkage disequilibrium analysis, the imputed loci rs28373093, rs1002205, rs1029421, rs2285647, and rs10235835, may represent independent and strong association signals. rs28373093, a polymorphism 1.5 kb upstream from the *ABCB1* transcription start site, has the strongest association. 2677 G>A/T and 3435C>T confer a clear gene-dosage effect on *ABCB1* mRNA expression. The systematic characterization of gene-wide common quantitative trait loci associated with *ABCB1* mRNA expression in normal liver tissues would provide the candidate markers to ABCB1-relevant clinical phenotypes in Chinese population.

## Introduction

The adenosine 5′-triphosphate-binding cassette transmembrane protein P-glycoprotein (P-gp, or multidrug resistance protein 1, MDR1) is encoded by human ATP-binding cassette, subfamily B, member 1 (*ABCB1*) gene, a well-studied membrane transporter [1]–[2]. P-gp acts an important role in maintaining cellular homeostasis, and is expressed in a range of tissues, including the apical pole of luminal epithelial cells of the stomach, the columnar epithelial cells of the small intestine, the colon, the biliary canaliculi of the liver, the brush border of both the proximal and distal renal tubules, the blood-tissue barrier sites and many types of tumors [3]–[7]. The list of substrates transported by P-gp includes a large number of structurally and functionally unrelated natural compounds and lipophilic xenobiotics. The extensive spectrum of substrates as well as the ubiquitous tissue distribution suggests P-gp as an efflux transporter with important roles in absorption, distribution and excretion of drugs, endogenous and exogenous chemicals, as well as the contribution to disease susceptibility [2]. P-gp overexpression in the barrier or excretory tissues protects the body from harmful compounds, and yet limits the drug access to target tissues. *ABCB1* gene, regulating the exposure to xenobiotics and carcinogens, is associated with the occurrences, development and histopathological features of a broad set of high-grade cancers, including renal epithelial tumors [8], colorectal cancer [9]–[10] and haematological malignancies [11]. Localized *ABCB1* overexpression also greatly contributes to the multidrug resistance, resulting in the low bioavailability of many therapeutic agents and unfavorable treatment outcomes for a variety of tumors, e.g. haematological malignancies [11]–[12]. For instance, the primary limitation to efficacy of the antineoplastic drug paclitaxel in drug-resistant cancer cells is the overexpression of the *ABCB1* gene [13].

Genetic variants in the human *ABCB1* gene have been widely studied to investigate the molecular basis of inter-individual variability in drug pharmacokinetic and pharmacodynamic response [14], such as bioavailability or clearance of drug probes [15], antidepressants [16], immunosuppressants [17], cholesterol lowering drugs [18], anti-retroviral agents [19], and anti-epileptic drugs [20]. In almost all investigations, two variants in the *ABCB1* coding sequence have been the intensively focused subjects: the nonsynonymous triallelic 2677G>A/T (ABCB1–893Ala>Ser/Thr) and the synonymous 3435C>T. T alleles of both variants have been more frequently associated with reduced *ABCB1* gene expression or functional activity [14]. However, discordant and even contradictory results were obtained in some studies using different tissue samples from diverse populations [21]–[22]. Subsequently, the feasibility of extrapolating a genotype-phenotype correlation to various drug administrations across study subjects is problematic. There were several notable limitations in the previous studies. Because of inappropriate phenotype definition, the perceptible phenotypic heterogeneity in samples confounded the detection of potential associations. Moreover, only a few variants with putative functional consequences were repeatedly examined, and most variants along the gene were underrepresented. Consequently, the functional significance of *ABCB1* polymorphisms to date has remained controversial, although extensive and intensive investigations have been conducted. In spite of the evident perception that genotype-dependent *ABCB1* expression alters the gene function and the susceptibility to human diseases, there has been little systematical scanning of genetic variants to establish the direct links between genetic effects and gene expression *in vivo*, and to understand the genetic basis of altered P-gp functional activity.

The present study interrogates cost-effective Tag single nucleotide polymorphisms (SNPs) spanning the whole *ABCB1* gene selected based on HapMap database, and in addition imputed more data that were not directly genotyped. We aimed to characterize *ABCB1* gene expression quantitative trait loci (eQTL) in normal liver tissue samples from Chinese population, and to identify the candidate variants as the future test targets correlated to clinical phenotypes in Chinese population.

## Materials and Methods

### Ethics statement

Written informed consent was received from all participants. Acquisition of all sample information from participants was approved by the Ethics Review Committee of the Chinese National Human Genome Center at Shanghai.

### Sample preparation

A total of 73 normal human liver specimens of unrelated Han Chinese transplant donors were collected from Tianjin First Center Hospital and stored in RNAlater (Sigma-Aldrich) at −80°C. Subject ages ranged from 30 to 45 years, and no drugs, alcohol and cigarettes were consumed for at least 30 days prior to surgery.

Genomic DNA and total RNA were simultaneously extracted from the liver tissue samples using QIAGEN AllPrep DNA/RNA Mini Kit according to the manufacturer's instruction. The quality of each RNA sample was assessed by the presence of strong 18S and 28S bands following agarose gel electrophoresis. Total RNA was treated with RNase-free DNase digestion to remove the residual genomic DNA. First-strand cDNA was synthesized using M-MLV Reverse Transcriptase (Promega) in a sterile 25 µl volume containing 0.5 µg of total RNA, 0.5 µM dNTP, 1 µM Oligo(dT)_15_, 5 µM random nonamer primers and 20 U ribonuclease inhibitor (Takara). The absence of genomic DNA in RNA samples was verified by the lack of PCR amplication products of negative controls without reverse transcriptase in reverse transcription reaction.

### Measurement of global mRNA expression level

To quantify *ABCB1* mRNA expression level, quantitative real-time PCR of cDNA was carried out using SYBR Green RT-PCR kit (Takara), and the following cycling conditions: initial pre-denaturation at 95°C for 30 s, and then 40 cycles of 95°C for 5 s, 60°C for 30 s. The PCR reactions were performed on ABI Prism 7900HT Real Time PCR System (Applied Biosystems, Inc., Foster City, CA). The β-actin house-keeping gene was used as an internal control. *ABCB1* primers were: (f) 5′-CAGGGAAAGTGCTGCTTGATG-3′ and (r) 5′-TCGATGAAGGCATGTATGTTGG-3′. The forward primer was designed to cross exons 26 and 27 to remove genomic DNA interference. β-actin primers were: (f) 5′-GTGACAGCAGTCGGTTGGAG-3′ and (r) 5′-AGGACTGGGCCATTCTCCTT-3′. Relative expression levels were calculated using standard curve method in program SDS2.0, and assays were performed in triplicate for all samples. Quantification of gene expression was presented as the ratio of ABCB1/β-actin.

### Allelic expression imbalance screening by SNaPshot genotyping

Allelic expression imbalance (AEI) was measured by parallel quantitative genotyping of heterozygous SNPs at the RNA level, and genomic DNA was used as a comparative reference to eliminate bias in favor of certain alleles. Three exonic SNP markers with high heterozygosity, namely 2677G>A/T, 3435C>T and rs3842, were selected as indicators for determining AEI levels. Three SNPs were amplified from both genomic DNA and cDNA templates, and PCR products were purified with exonuclease I and shrimp alkaline phosphatase. To discriminate alleles, SNaPshot extension reactions to add a single fluorophore-labeled dideoxyribonucleosied triphosphate were carried out using ABI PRISM SNaPshot Multiplex Kit (Applied Biosystems, Inc., Foster City, CA). Extension products were electrophoresed on ABI PRISM 3730XL DNA Analyzer. Genotype calling were carried out using GeneMapper V4.0 software. The collected fluorescence intensity is proportional to the amount of amplified alleles. AEI was measured as the allelic expression ratio, calculated by dividing two allelic ratios of fluorescence signals for cDNA by the ratio for genomic DNA in heterozygous samples to calibrate the inherent bias incurred by fluorescent labels. Each allelic expression ratio was independently measured in triplicate.

### Loci selection and genotyping

The pairwise tagging algorithm in Haploview 4.2 software [23] was used to select a set of Tag SNPs by using the criteria of an linkage disequilibrium (LD) cutoff *r* ^2^ = 0.8 and a minor allele frequency (MAF) greater than 0.1 based on HapMap Phase III data (release #27). A total of 35 Tag SNPs captured common variants spanning an approximate 290 kb region ranging from 40 kb upstream to 40 kb downstream of the *ABCB1* gene. Tag SNPs genotyping was performed using SNaPshot method or by direct sequencing using BigDye Terminator v3.1 Cycle Sequencing Kit (Applied Biosystems, Inc., Foster City, CA) on 3730XL sequencer.

### Statistical analysis

Data summary and association statistics of Tag SNPs were made using Plink V1.07 software [24]. Univariate linear regression analysis was conducted to assess the association between Tag SNPs and *ABCB1* gene expression assuming an additive model, in which the genotype of each SNP was coded as 0, 1 or 2 corresponding to the counts of the minor allele in each genotype. MAF lower than 10% were not included in the linear regression analysis. In the linear regression model to test for the association of triallelic 2677G>A/T with the normalized ABCB1 expression, the 2677G allele was combined with A allele in the linear regression analysis, which compared gene expression between the integrated genotypes and the genotypes with T allele, because the 2677T allele has been more reproducibly associated with reduced gene expression or deficient P-gp activity [14]. Bonferroni and FDR correction were used to adjust for multiple test results. In addition, genotype imputation for a 500 kb stretch across the entire *ABCB1* gene and flanking regions was carried out by IMPUTE V2.1.2 [25] using two independent multi-population reference panels [26]: 1000 Genomes Phase I (interim) and HapMap Phase III release 2 datasets. More detailed option-settings for IMPUTE are listed in Table S1. The association strength of imputed loci was evaluated with Bayesian regression analysis in the program BIMBAM by computing Bayes factors (BFs) [27]–[28]. We specified the priors for additive effects σ_a_ = 0.05, 0.1, 0.2, 0.4 and the priors for dominant effects σ_d_ = σ_a_/4. P values for each imputed SNP were produced by 100,000 random permutations to assess the significance of the BFs obtained. Given the advantages of Bayesian method, multi-SNP analyses were also carried out to estimate the association of SNP combinations. LD estimation and plot imaging were conducted using an R-based package snp.plotter [29]. Genotypes obtained from the imputation are probability distributions instead of hard genotype calls, and therefore, these data cannot be directly used to perform LD analysis. The genotype data of associated loci in Chinese Han were extracted from 1000 Genomes datasets for LD analyses. Haplotypes were reconstructed with fastPHASE software [30].

## Results

### Mapping quantitative trait loci associated with *ABCB1* mRNA expression

*ABCB1* mRNA expression levels varied by nearly three folds in our samples, and showed an approximately normal distribution (Figure S1). A total of 35 Tag SNPs were genotyped for 73 normal liver samples, including the intensively studied polymorphisms rs3213619 (−129T>C), rs1128503 (1236C>T), rs2032582 (2677G>A/T) and rs1045642 (3435C>T). Genotyping results for all Tag SNPs are given in Table S2. Via the linear regression, we observed ten loci suggestive of associations (*P*<0.05) among the Tag SNPs (Table S3, Figure S2). Seven loci (rs1882478, 3435C>T, rs1922243, rs2373588, rs4148738, rs12535512, and rs1978095) still showed significant associations with *ABCB1* gene expression after Bonferroni correction. Almost every associated locus accounted for a respectable contribution to variation in gene expression for R^2^>10% from the linear regression. Both 2677G>A/T (R2 = 0.111, P<0.004) and 3435C>T (R2 = 0.12, P = 0.0026) also had significant associations in these normal liver samples (Table S3). The T allele of the two loci is associated with lower gene expression (Figure S3). The gene expression level is the lowest for TT genotype of 2677G>A/T, and lower for AT and GT genotype compared to GA and GG (Figure S3).

**Figure 1:**
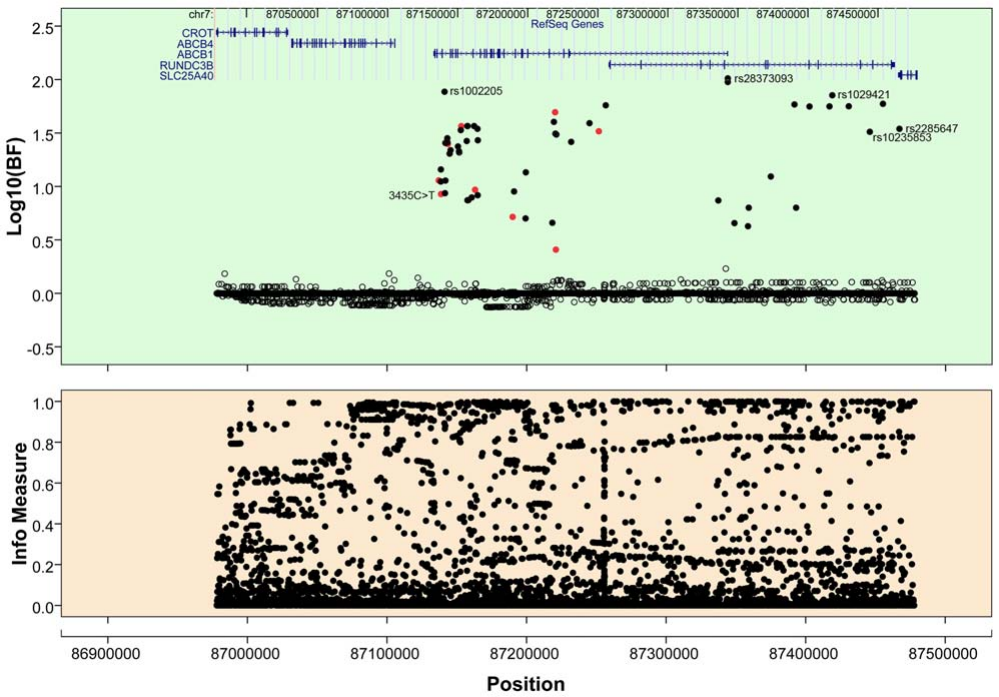
Association results of imputed loci using 1000 Genome datasets as reference panel. The upper panel illustrates log-transformed BFs of single SNPs with Bayesian regression. The black dots are the imputed loci showing the associations with *ABCB1* gene expression. The red dots represent Tag SNPs with significant associations. The lower panel indicates the infor measures of imputed loci. Physical coordinates in the figure are based on Human Reference Genome Sequence Build 37.

Furthermore, we performed imputation in a range of 500 kb around *ABCB1* gene using 1000 Genomes Phase I and Hapmap III datasets separately as reference panels. The triallelic SNP 2677G>A/T was removed during the imputation in that it is biallelic in the reference datasets. We imputed 5651 loci (including Tag SNPs) using 1000 Genomes datasets and tested the associations of imputed loci with Bayesian regression analysis (Fig. 1). More loci were identified with greater strength of association than Tag SNPs, and the information metrics (infor>0.6) indicate that the associated SNPs were imputed with a high degree of certainty (Table 1). rs28273093/rs35901799, a dinucleotide polymorphism 1.5 kb upstream from the transcription start site, had the strongest association (log_10_(BF)>2, *P* = 4.00E-5) with the gene expression. In the upstream region of the *ABCB1* gene, a few SNPs were imputed and identified with significant associations. While, no Tag SNPs were detected that showed significant associations with the gene expression in this region. A total of 361 loci were imputed within the same range using Hapmap III datasets (Figure S4). A concordant group of associations was produced after Bayesian regression analysis, and all associated loci from Hapmap III are included in the result from 1000 Genomes datasets (Table S4).

**Table 1:** Association with ABCB1 gene expression of imputed loci with top-ranked BFs using 1000 Genome datasets as reference panels.

SNP	Position	A1 A2	Infor(Impute)	MAF	Log10(BF)	P Value
**rs28373093**	**87344023**	**C G**	**0.654**	**0.423**	**2.011**	**4.00E-05**
rs35901799	87344022	C T	0.658	0.427	1.977	4.00E-05
**rs1002205**	**87141174**	**C G**	**0.893**	**0.402**	**1.886**	**8.00E-05**
**rs1029421**	**87418861**	**C T**	**0.888**	**0.48**	**1.853**	**1.30E-04**
rs6966166	87455155	T C	0.919	0.46	1.773	1.60E-04
rs7779623	87391761	G A	0.925	0.463	1.767	1.50E-04
rs34800935	87256602	C T	0.726	0.399	1.759	1.10E-04
rs12704370	87430669	A G	0.928	0.462	1.75	1.70E-04
rs7793933	87416839	T C	0.927	0.462	1.749	1.70E-04
rs6465119	87402536	G A	0.915	0.452	1.748	2.10E-04
rs12535512*	87220334	T C		0.445	1.694	1.90E-04
rs13229143	87219481	G C	0.959	0.444	1.604	3.40E-04
rs13233308	87244960	C T	0.978	0.455	1.592	3.20E-04
rs2373587	87157466	G C	0.996	0.391	1.565	3.50E-04
rs2373585	87157706	T C	0.996	0.391	1.565	3.50E-04
rs10236274	87162341	G A	0.997	0.391	1.565	3.60E-04
rs2373588*	87153160	A G		0.39	1.564	3.30E-04
rs7787569	87164756	A C	0.994	0.392	1.539	3.60E-04
**rs2285647**	**87466971**	**A G**	**0.851**	**0.225**	**1.539**	**1.60E-04**
rs2141849	87152790	A C	0.988	0.394	1.527	3.80E-04
rs1978095*	87251641	G A		0.233	1.516	3.40E-04
**rs10235853**	**87445748**	**G T**	**0.698**	**0.467**	**1.511**	**2.10E-04**
rs2188526	87220562	C T	0.982	0.455	1.494	4.70E-04
rs1858923	87221216	A G	0.98	0.456	1.486	4.90E-04
rs4148751	87143153	C T	0.985	0.392	1.451	4.10E-04
rs10248420	87164986	G A	0.988	0.415	1.432	5.20E-04
rs7787082	87157051	A G	0.979	0.415	1.425	5.20E-04
rs12720464	87231853	T C	0.977	0.226	1.417	3.60E-04
rs4148750	87143275	C T	0.986	0.393	1.412	4.80E-04
rs17149699	87141751	T C	0.98	0.393	1.407	4.60E-04
rs1922243*	87143504	T C		0.39	1.397	5.10E-04
rs4148744	87150774	A G	0.989	0.399	1.374	4.40E-04
rs1922244	87145483	A G	0.986	0.399	1.339	5.30E-04
rs1882477	87151524	G C	0.974	0.415	1.328	6.40E-04
rs2373589	87151658	T C	0.977	0.413	1.318	6.60E-04
rs7779562	87144816	C G	0.986	0.4	1.307	6.20E-04
rs2235047	87138532	C A	0.943	0.38	1.159	1.16E-03
rs2235018	87199365	C T	0.933	0.211	1.132	8.60E-04
rs117647666	87374828	A G	0.923	0.208	1.093	1.09E-03
rs1882478*	87137018	C T		0.466	1.056	1.44E-03
rs6949448	87141814	C T	0.88	0.49	1.055	1.43E-03
rs2235048	87138511	G A	0.961	0.424	1.046	1.77E-03
rs4148738*	87163049	C T		0.432	0.969	1.93E-03
rs1045642*	**87138645**	**A G**		**0.411**	**0.929**	**2.63E-03**
rs868755*	87189930	T C		0.452	0.715	5.55E-03
rs3789243*	87220886	A G		0.39	0.409	2.05E-02

*P* values were produced by 100000 random permutations. The SNPs marked with asterisks denote Tag SNPs. The SNPs representing independent strong associations are in bold. Physical positions of the SNPs are based on Human Reference Genome Sequence Build 37.

### LD analyses of to elucidate the independent associations

**Figure 2:**
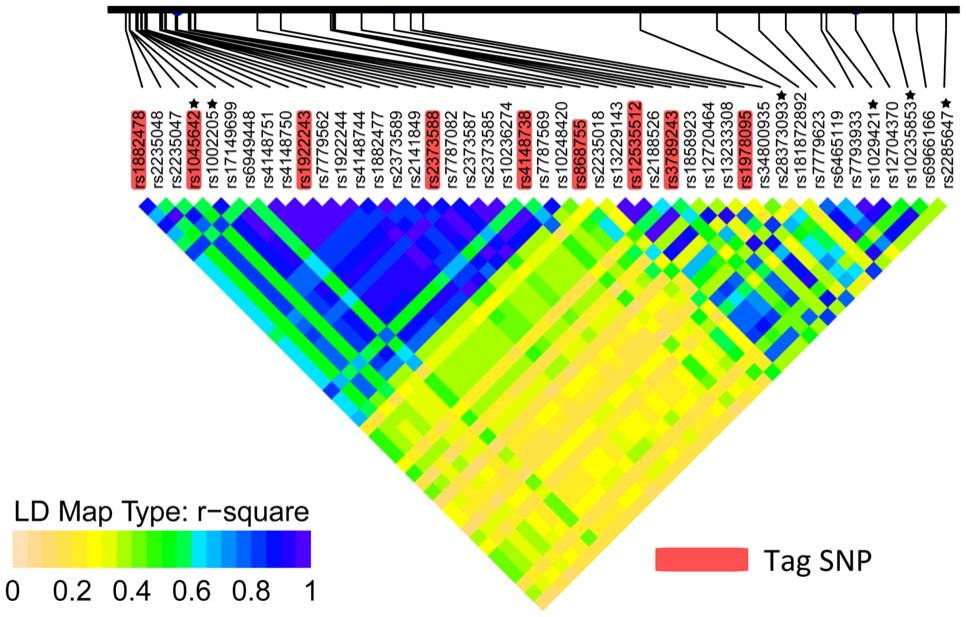
LD plot of SNPs with top-ranked BFs in CHB of 1000 Genome Phase I. The colors indicate the strength of pairwise LD according to *r* ^2^ metrics. The SNPs marked with asterisks represent independent strong associations. Tag SNPs are shadowed in pink.

LD analysis of Tag SNPs indicates that these SNPs are overall not in strong linkage disequilibrium (Figure S2). Therefore, Tag SNPs with significant associations probably represent several independent association signals. The collaborative effects of multiple loci are likely to contribute to the *ABCB1* gene expression. We next extracted the data of associated loci in Chinese Han from 1000 Genomes Phase I for LD analysis. The LD pattern (Fig. 2) in Chinese Han of Beijing (CHB) illustrates that although some SNPs are in strong LD, and the associated loci may represent multiple independent associations, which is consistent with the results from Tag SNPs. The pairwise LD in CHB is similar to, but stronger than that in Chinese Han of South (CHS) (Figure S5, Figure S6). The locus with the strongest association, rs28373093, is not tagged by Tag SNPs, for the pairwise *r* ^2^ are below 0.6 for all Tag SNPs. Moreover, the imputed loci with stronger associations, rs1002205 (log_10_(BF) = 1.886, P = 8.00E-5), rs1029421 (log_10_(BF) = 1.853, P = 1.30E-4) and rs2285647 (log_10_(BF) = 1.539, P = 1.60E-4), were identified representing the independent association signals, explaining the proxy associations of the Tag SNP rs2373588, rs12535512 and rs1978095, respectively. rs10235853 (log_10_(BF) = 1.511, P = 2.10E-4) is not in LD with other loci showing a stronger association.

### Bayesian multi-SNP analyses indicating the combined effects of multiple independent variants

**Figure 3:**
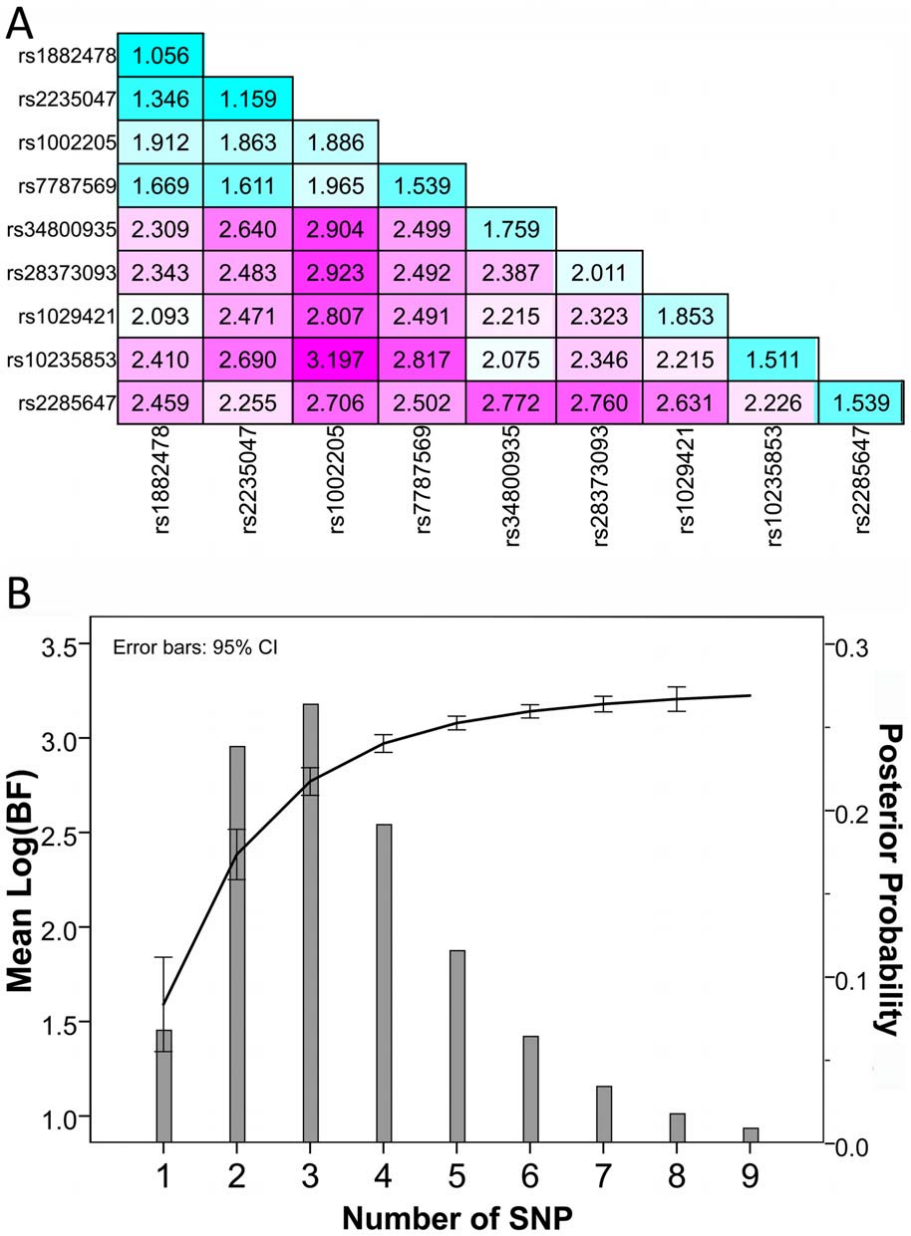
Multi-SNP results of Bayesian analyses. (A) BF values of pairwise loci combinations in Multi-SNP analyses. The colors are corresponding to BFs. (B) BF values and posterior probabilities for different multi-SNP combinations. Means of BFs corresponding to different SNP combinations are charted in line, and means of posterior probabilities in bars.

Following the results of the association analyses of single locus and LD analyses, analyses of multiple loci were performed using Bayesian method. A total of nine SNPs (rs1882478, rs2235047, rs1002205, rs7787569, rs34800935, rs28373093, rs1029421, rs10235853, and rs2285647) were included in the multi-loci analyses. These SNPs include the five loci of independent associations. BF values of pairwise loci combinations are all higher than those of single locus (Fig. 3A). A pairwise model of rs1002205 and rs10235853 produces the highest BF (log_10_(BF) = 3.197). We observed that BF values increase with the number combined loci of the combinations, and the values sharply rises up to the combination of five loci (Fig. 3B). Posterior probabilities for multi-SNP combinations, up to the 5-SNP model, also surpass the posterior probability for single locus, which is less than 7%, and the posterior probability for three loci hits the highest, exceeding 26% (Fig. 3B). Hence, the association of multiple loci with *ABCB1* gene expression is stronger than that of single locus, and the combined effects of multiple SNPs contribute to *ABCB1* gene expression.

### Allelic imbalance in *ABCB1* gene expression

**Figure 4:**
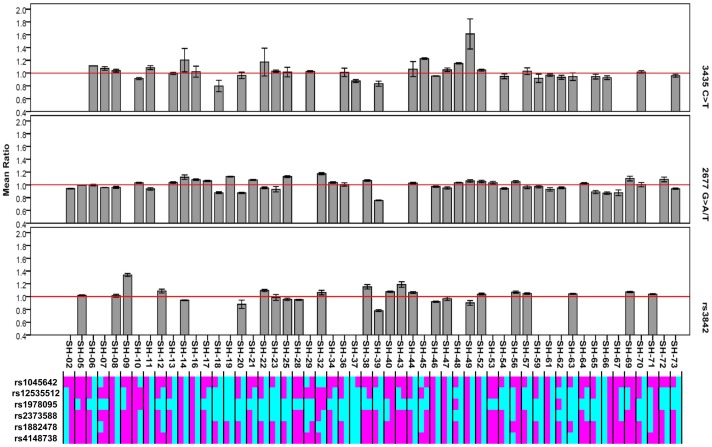
Allele specific *ABCB1* mRNA expression in normal liver samples and the phases of Tag SNPs with significant associations. In the upper panel, allelic expression ratios of samples heterozygous for at least one marker SNP are presented by bar plots. Each allelic expression ratio was independently measured in triplicate using SNaPshot. In the lower panel, each pair of columns represents the two phases of associated Tag SNPs in each sample heterozygous for marker SNP. Alleles associated with higher gene expression are denoted in pink, and alleles associated with reduced expression in cyan.

To further evaluate *cis*-regulatory influences on *ABCB1* gene expression, we examined differences in the relative expression of both allelic transcripts. Out of our samples, 54 subjects were heterozygous for at least one of the three marker SNPs indicating AEI (Fig. 4). However, the relative allelic expression does not provide a clear clue to *cis*-acting effects on *ABCB1* gene expression. A uniform deviation of allelic expression ratios from the allelic transcript equality, which would suggest the *cis*-acting function of 2677G>A/T and 3435C>T, was not observed for heterozygotes of these two loci. This is inconsistent with previous findings [31]–[32]. In the light of multiple SNPs contributing to the gene expression, we estimated the haplotypes of Tag SNPs that remain significant after Bonferroni correction. Almost every haplotype per individual is made up of alleles with positive and negative effects, which are associated with high and low gene expression (Fig. 4). In contrast to single locus, the interplay between different alleles of multiple functional loci blurs the actual effects of individual loci. Consequently, the relative allelic expression ratios do not appear as expected.

### Contribution of 2677–3435 to *ABCB1* gene expression

**Figure 5:**
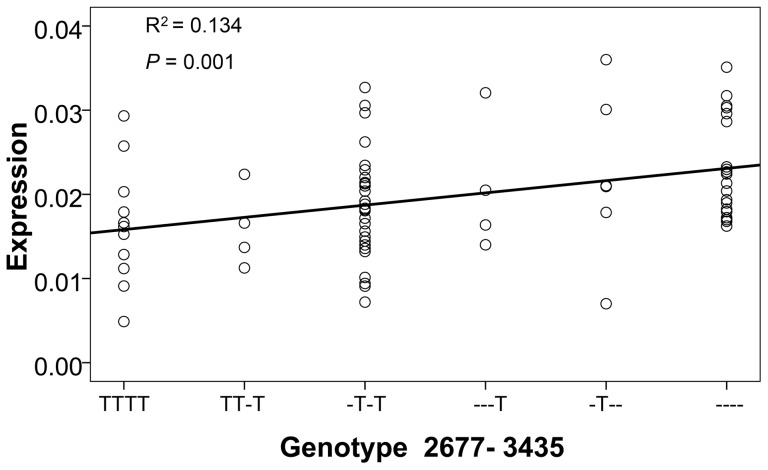
Relationship between *ABCB1* mRNA expression and the genotype combinations of 2677G>A/T and 3435C>T. The increase of T alleles at both loci is associated with reduced *ABCB1* gene expression.

The two SNPs, 2677G>A/T and 3435C>T, were paid considerable attention to when investigating the association of *ABCB1* gene with the gene expression, the transporter activity, and various clinical phenotypes. We also identified the associations of them with the gene expression, and the correlation of T allele with reduced gene expression. 2677G>A/T and 3435C>T have exhibited the most reproducible association with the gene expression, which was attributed to the strong LD between both loci [33]. Actually, it is difficult to accurately estimate the LD for triallelic 2677G>A/T. We grouped the combinations of genotypes according to T allele status at each locus, and performed general linear regression to analyze the contribution of two loci to *ABCB1* gene expression (Fig. 5). We found that the more T alleles present at two loci, the lower *ABCB1* gene expression is (*P* = 0.001). Therefore, it is suggested that the independent effects from 2677G>A/T and 3435C>T contribute to the gene expression.

## Discussion

In present study, we adopted Tag SNP strategy to capture most common variants of the *ABCB1* gene, and performed imputation to estimate more genotypes of directly untyped variants within the region of interest using two separate reference panels. We accessed the strength of association by computing BFs, and more finely mapped common eQTLs within a 500 kb region across the *ABCB1* gene. Dozens of loci showed evidence of association with *ABCB1* gene expression, although some are in strong LD, thus representing identical association signals. LD analysis (Fig. 2) illustrates that rs28373093, rs1002205, rs1029421, rs2285647, and rs10235853 may stand for independent associations with strong evidence. This is consistent with the results from multi-loci analyses, in which the posterior probabilities for multi-SNP combinations, up to the 5-SNP model, exceed that for single locus (Fig. 3B). A dinucleotide polymorphism was assigned as three SNPs, namely, rs28373093, rs35901799, and rs28656907, in the proximal promoter region. This polymorphism have the strongest evidence of association in present study, and was also associated with *ABCB1* gene expression in the lymphoblastoid B cell lines from the Centre d'Etude du Polymorphisme Humain collection [34]. In a clinic study of depression [35], a set of SNPs in the *ABCB1* gene were reported to predict the response to antidepressant treatment in depressed patients receiving drugs of P-gp substrates, including rs7787082 and rs10248420, which were also identified in the present study (Table 1) and are in strong LD with rs1002205 (Fig. 2). Accordingly, there may be shared variants affecting *ABCB1* gene expression and function in both the central nervous system and liver tissue. In epilepsy patients of Han Chinese [36], rs3789243, as Tag SNP, was also identified to be associated with drug resistance, but not with *ABCB1* mRNA levels in the brain samples, despite a relatively weak association evidence in liver tissue (Table S3).

The associated SNPs primarily distribute in intragenic and upstream regions of *ABCB1* gene. No association was identified in the downstream region, in accordance with previous reports. The upstream is largely invariant as previously reported [37], and no Tag SNPs in this region are significantly associated with the gene expression. However, rs28373093, rs1029421 and rs2285647 were imputed and present the strong associations. A variant upstream to the translation start site, rs3213619 (−129T>C), has been frequently reported to be associated with *ABCB1* gene expression. *ABCB1* −129C showed association with the reduced mRNA expression in both colorectal adenocarcinomas and adjacent noncancerous colorectal tissues in Japanese subjects [38]. Similarly, the placentas of Japanese women carrying the −129C allele had less P-gp expression [39]. The −129T>C combined with 2677G>T and 3435C>T contributes to the P-gp expression in renal transplant recipients [40]. Compared with the allele frequency in these reports, −129T>C was too rare in our samples to estimate its impact on *ABCB1* gene expression. Notwithstanding, multiple variants in the upstream region were suggested to have separate effects on the gene expression.

Although large-scale association studies are becoming more common following rapid advances in cost-effective and high-throughput genotyping technologies, most ongoing association studies still genotype a proportion of SNPs throughout the whole genome or in a set of candidate genomic regions of interest. Imputation-based analysis can allow higher-density variants to be tested for association with relevant traits, thus boosting the statistical power of study and facilitating fine-mapping of the associations [41]. In the present study, two independent reference panels, recently released 1000 Genome Phase I and HapMap Phase III data, were used for imputation. Concordant results were obtained from both reference panels. All associated SNPs imputed from HapMap reference are included in those from 1000 Genome data. Hence, the results confirm each other. Prominent advantages of Bayesian approach over classical statistical methods lie in its robustness to factors that affect statistical power, such as sample size or minor allele frequency, flexibility in analysis models, and better comparability and interpretability across different sets of results [42]. Analyses of combinations of multiple associated SNPs were carried out using Bayesian method. The stronger association with *ABCB1* gene expression, and the higher posterior probability of multi-loci combinations than of single SNP, further supports multiple SNPs with independent effects contributing to the gene expression. The BFs of SNPs with evidence of association in the present study are relatively lower than those reported in previous studies at genome-wide scale [43]–[44]. Probably, the collaborative contribution of multiple functional SNPs to the gene expression leads to an underestimation of the association strength.

**Figure 6:**
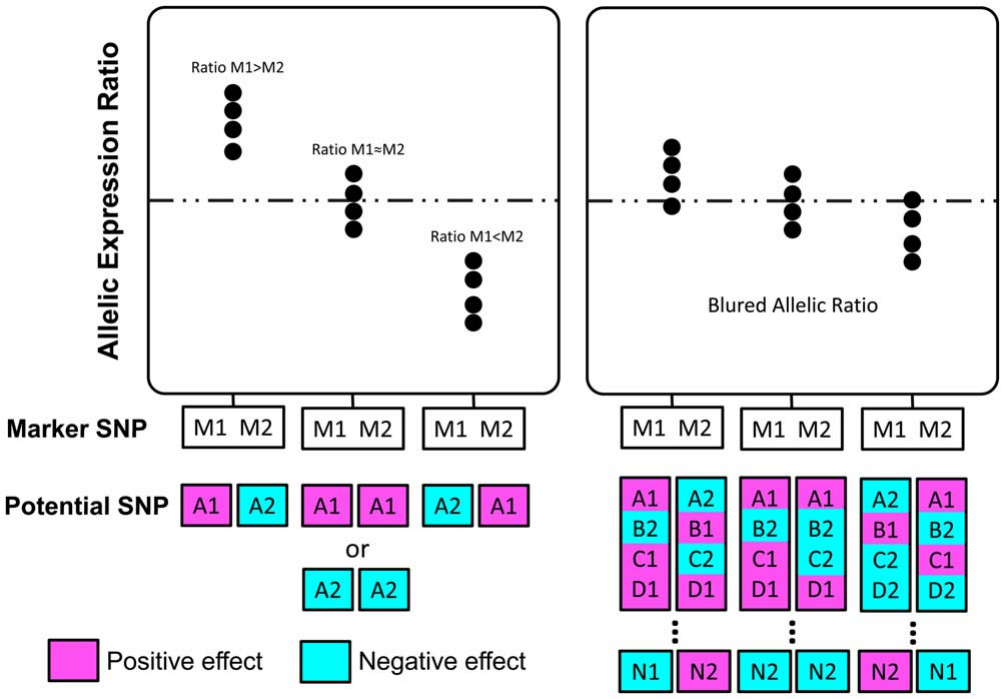
Allelic expression ratios resulted from collaborative effects of different alleles of multiple functional SNPs. Allele M1 and M2 of the marker SNP indicating AEI correspond to the alleles of single *cis*-acting SNP or the haplotypes consisting of multiple *cis*-acting SNPs which are in the same phase with M1 and M2. Positive-effect alleles associated with high gene expression are denoted in pink, and negative-effect alleles associated with low expression are denoted in cyan. In the left panel, allelic expression ratios clearly indicate the effect of single *cis*-acting SNP. In the right panel, allelic expression ratios are blurred by multiple *cis*-acting effects.

We attempted to identify the clue of *cis*-acting factors regulating *ABCB1* gene expression through AEI, as differential allelic expression was reported between alleles of 2677G>A/T and 3435C>T [31]–[32]. Three markers, 2677G>A/T, 3435C>T, and rs3842, provide rich information to indicate the relative expression of both alleles (Fig. 4). Unfortunately, no clear evidence was found to support *cis*-acting regulation on *ABCB1* gene expression. In particular, allelic expression ratio of 2677G>A/T and 3435C>T does not suggest this. Paradoxically, each allelic expression ratio is the synthetical outcome of interaction among different alleles at individual functional loci that are not in strong LD with each other, as alleles of marker SNPs may be in the same phase consisting of opposite-effect alleles of functional loci (Fig. 6). As a result, the allelic expression ratios do not reflect the true effects of each *cis*-acting factor. Although investigating *cis*-acting elements through differential allelic expression prevents interference from *trans*-acting regulators or environmental confounders and improves the sensitivity to *cis*-acting effects even using limited sample size [45]–[46], the power of this strategy is attenuated if multiple *cis*-acting factors act simultaneously, owing to an inability to distinguish the effects of individual *cis*-acting elements.

Almost all research on the *ABCB1* gene has focused on two variants, 2677G>A/T and 3435C>T, but without reaching a convincing conclusion. Previous studies proposed that the strong LD among 1236C>T, 2677G>A/T, and 3435C>T resulted in their associations with *ABCB1* expression and clinical phenotypes [33]. However, this is not the case in the present study. In practice, a strong LD does not exist between 1236C>T and 3435C>T in our samples and Chinese Han from Beijing of HapMap. LD between triallelic 2677G>A/T and other polymorphisms is not strong. In addition, the increased number of T allele at both loci is significantly associated with the reduction of gene expression (Fig. 5). A clear gene-dosage effect on *ABCB1* mRNA expression is observed in normal liver tissues. However, this trend contradicts reports that T alleles of two loci were associated with higher expression in kidney cortex in genotype and diplotype analyses [47]. Yet, the placentas from mothers homozygous for both 2677T and 3435T allele (TT/TT) showed significantly lower P-gp expression versus mothers with the GG/CC genotype constellation [48]. Anyway, it is difficult to attribute the association of both variants to their LD, and their cumulative effects should play a part in *ABCB1* gene expression.

In conclusion, the independent effects of multiple functional SNPs contribute to *ABCB1* gene expression in normal liver tissues from Chinese population. A dinucleotide polymorphism, rs28373093, 1.5 kb upstream from the transcription start site, was identified to have the strongest association with the gene expression. The imputed loci rs1002205, rs1029421 and rs2285647, and rs10235835, represent separate association signals. 2677G>A/T and 3435C>T may play their respective roles in *ABCB1* expression. This comprehensive characterization of eQTLs in the *ABCB1* gene in normal liver tissues could, therefore, provide predictors of ABCB1 function-relevant phenotypes in Chinese population.

## Supporting Information

Figure S1 **Frequency distribution of** ***ABCB1*** **gene expression in 73 normal liver samples.** *ABCB1* gene expression levels were determined by relative quantitative RT-PCR and normalized to β-actin.
(TIF)

Figure S2 **Association results and pairwise LD plot of Tag SNPs.** The upper part illustrates the log-transformed *P* values in linear regression analysis. The SNPs with *P* value<0.05 are shown in red dots. LD map is plotted based on *r* ^2^ metrics, and the colors correspond to the strength of pairwise LD.
(TIF)

Figure S3 ***ABCB1*** **gene expression levels with different genotypes of associated Tag SNPs in normal liver samples.** *ABCB1* gene expression levels represent the relative ratios of ABCB1 to β-actin mRNA.
(TIF)

Figure S4 **Association results of imputed loci using HapMap III datasets as reference panel.** The upper panel illustrates log-transformed BFs of single SNP with Bayesian regression. The black dots are the imputed loci showing the associations with *ABCB1* gene expression. The red dots represent Tag SNPs with a significant association. The lower panel denotes the infor measures of imputed loci. Physical coordinates in the figure are based on Human Reference Genome Sequence Build 36.
(TIF)

Figure S5 **LD plot of SNPs with top-ranked BFs in CHS of 1000 Genome Phase I.** The colors indicate the strength of pairwise LD according to *r* ^2^ metrics.
(TIF)

Figure S6 **LD plot of SNPs with top-ranked BFs in combined Chinese Han (CHB and CHS) of 1000 Genome Phase I.** The colors indicate the strength of pairwise LD according to *r* ^2^ metrics.
(TIF)

Table S1 **Reference panels and detailed option-settings used in IMPUTE.**
(DOC)

Table S2 **Genotyping results of all Tag SNPs.**
(DOC)

Table S3 **Tag SNP association with** ***ABCB1*** **gene expression using linear regression analysis.** Physical positions of the SNPs are based on Human Reference Genome Sequence Build 36. Nominal significant *P* values<0.05 are shown in italics.
(DOC)

Table S4 **Association results of imputed loci with top-ranked BFs using HapMap III datasets as reference panel.** SNPs marked with an asterisk denote Tag SNPs. *P* values were produced by 100000 random permutations. Physical positions of the SNPs are based on Human Reference Genome Sequence Build 36.
(DOC)
