# Supplementary figures and images for: Gene-Wide Characterization of Common Quantitative Trait Loci for ABCB1 mRNA Expression in Normal Liver Tissues in the Chinese Population
Source: PLoS One. 2012 Sep 26;7(9):e46295. doi: 10.1371/journal.pone.0046295 (PMC3458811; doi:10.1371/journal.pone.0046295)

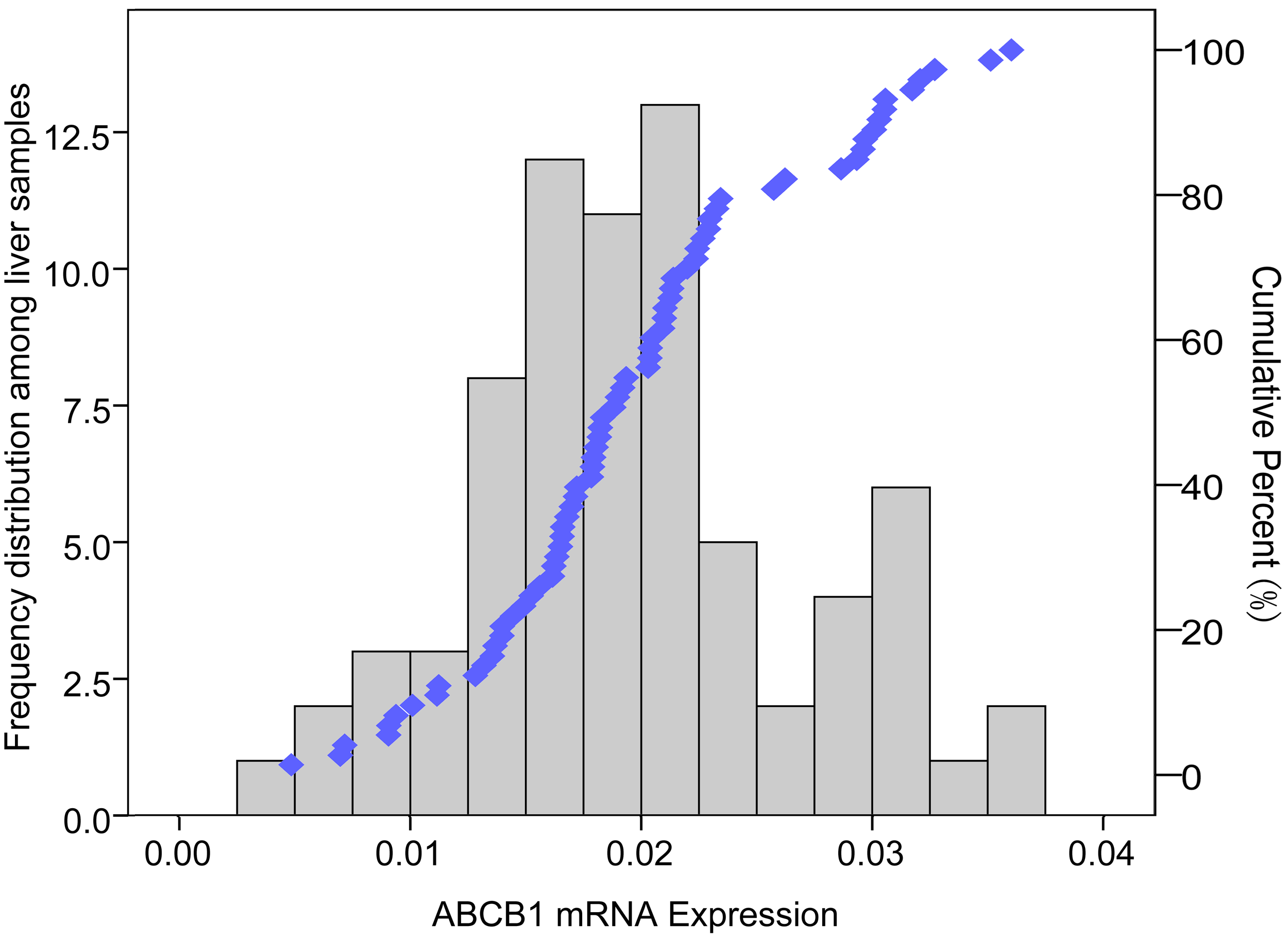

Supplement: Figure S1 — Frequency distribution of ABCB1 gene expression in 73 normal liver samples. ABCB1 gene expression levels were determined by relative quantitative RT-PCR and normalized to β-actin. (TIF) [file pone.0046295.s001.tif]

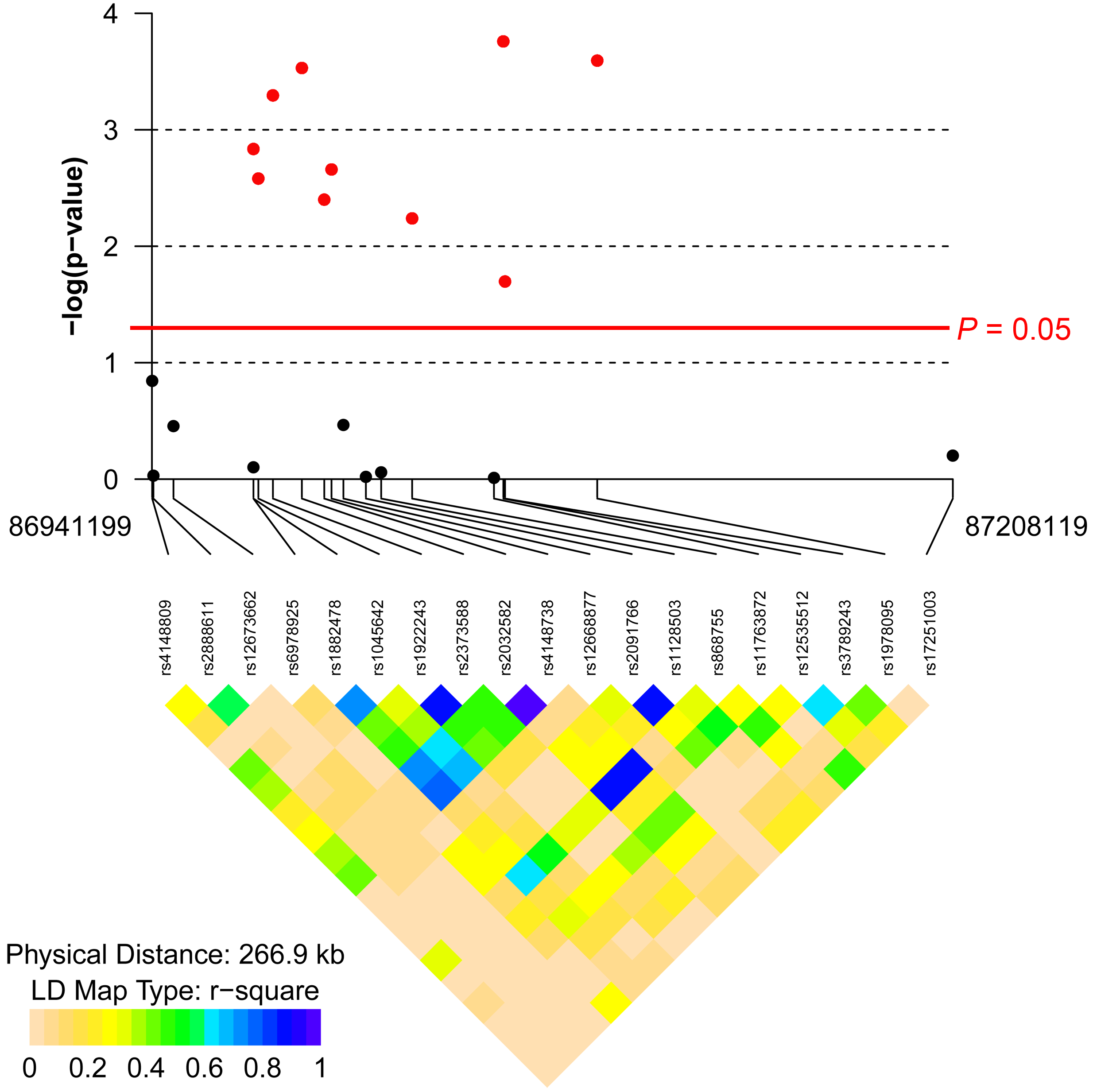

Supplement: Figure S2 — Association results and pairwise LD plot of Tag SNPs. The upper part illustrates the log-transformed P values in linear regression analysis. The SNPs with P value<0.05 are shown in red dots. LD map is plotted based on r 2 metrics, and the colors correspond to the strength of pairwise LD. (TIF) [file pone.0046295.s002.tif]

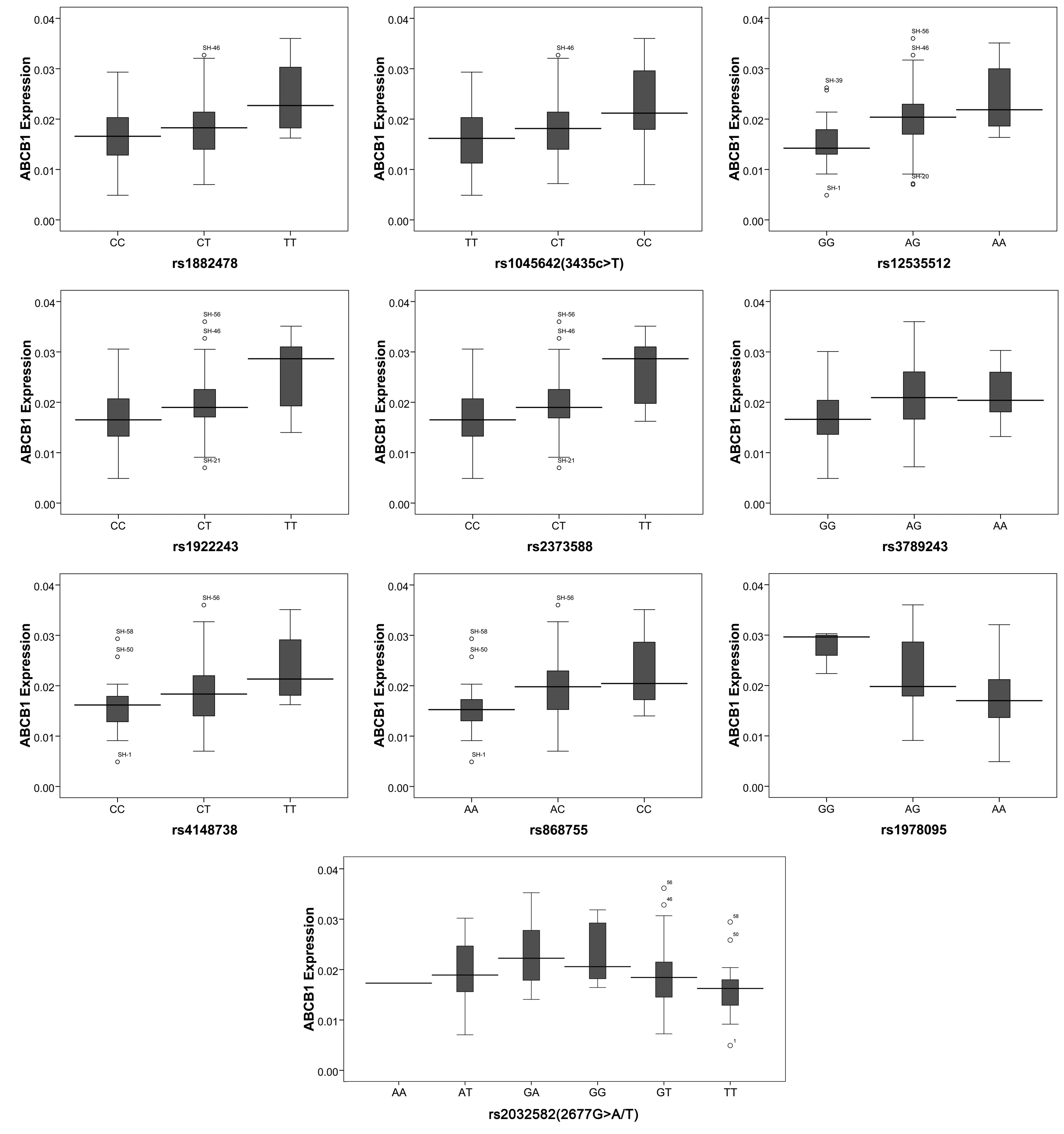

Supplement: Figure S3 — ABCB1 gene expression levels with different genotypes of associated Tag SNPs in normal liver samples. ABCB1 gene expression levels represent the relative ratios of ABCB1 to β-actin mRNA. (TIF) [file pone.0046295.s003.tif]

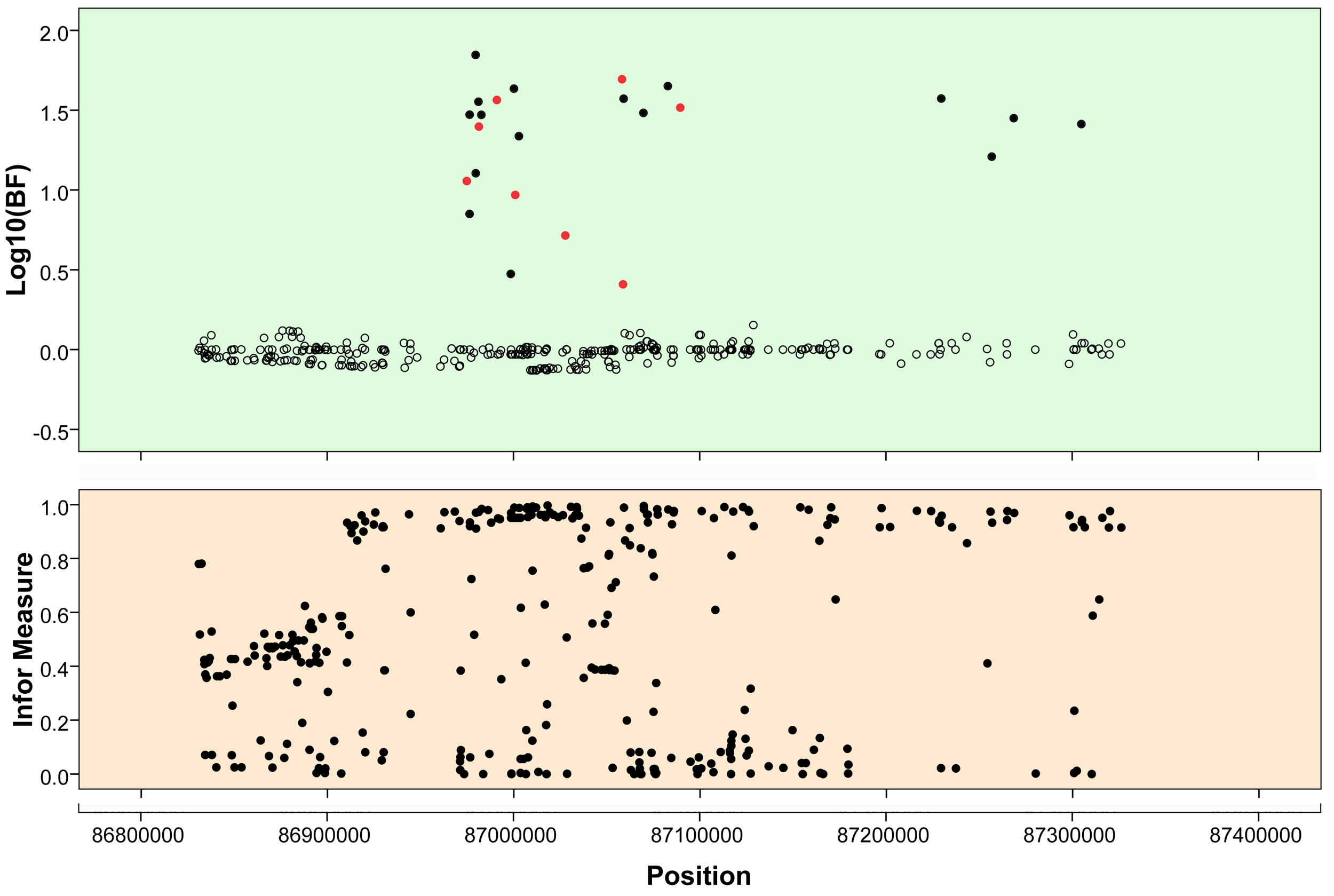

Supplement: Figure S4 — Association results of imputed loci using HapMap III datasets as reference panel. The upper panel illustrates log-transformed BFs of single SNP with Bayesian regression. The black dots are the imputed loci showing the associations with ABCB1 gene expression. The red dots represent Tag SNPs with a significant association. The lower panel denotes the infor measures of imputed loci. Physical coordinates in the figure are based on Human Reference Genome Sequence Build 36. (TIF) [file pone.0046295.s004.tif]

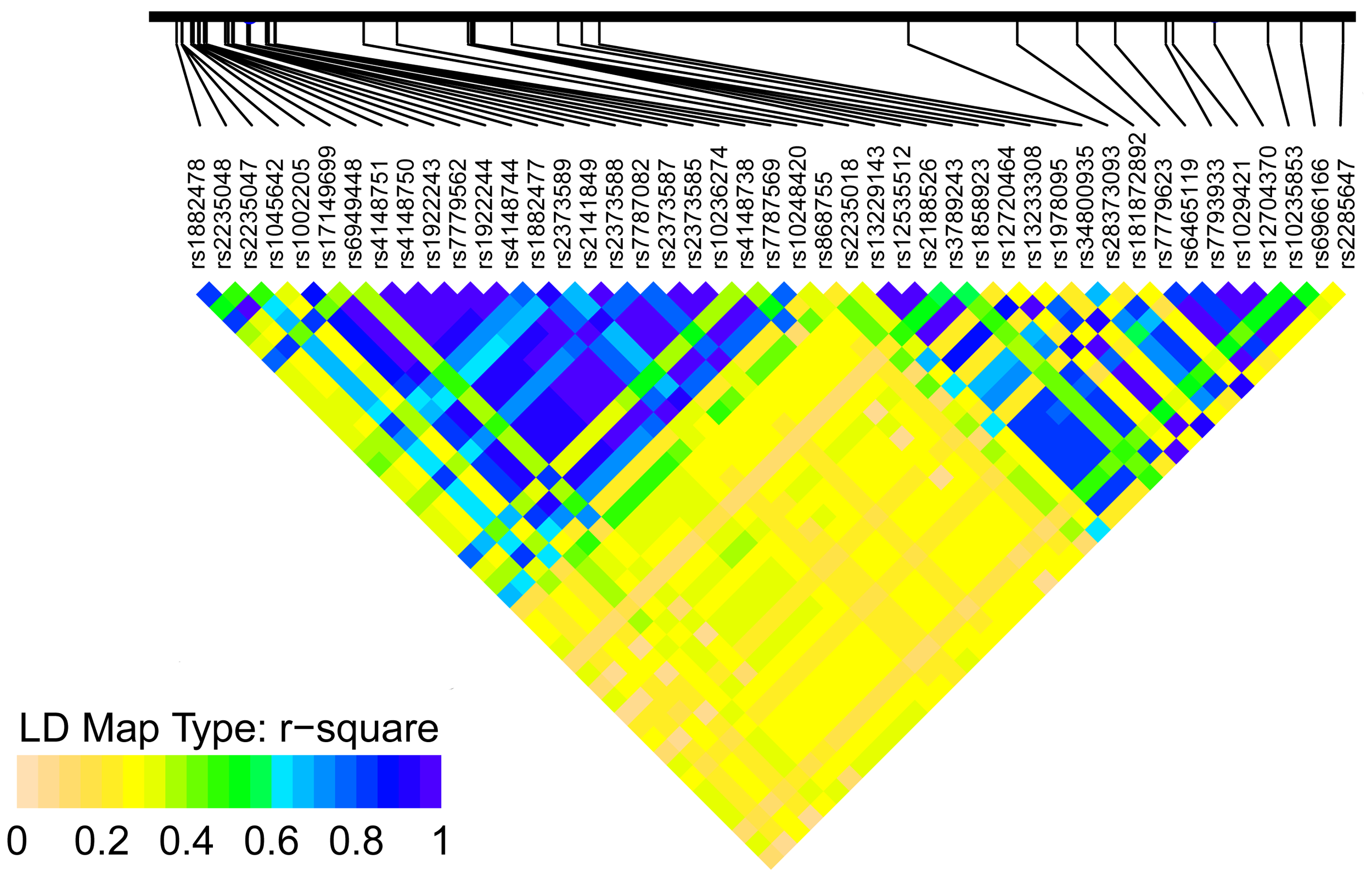

Supplement: Figure S5 — LD plot of SNPs with top-ranked BFs in CHS of 1000 Genome Phase I. The colors indicate the strength of pairwise LD according to r 2 metrics. (TIF) [file pone.0046295.s005.tif]

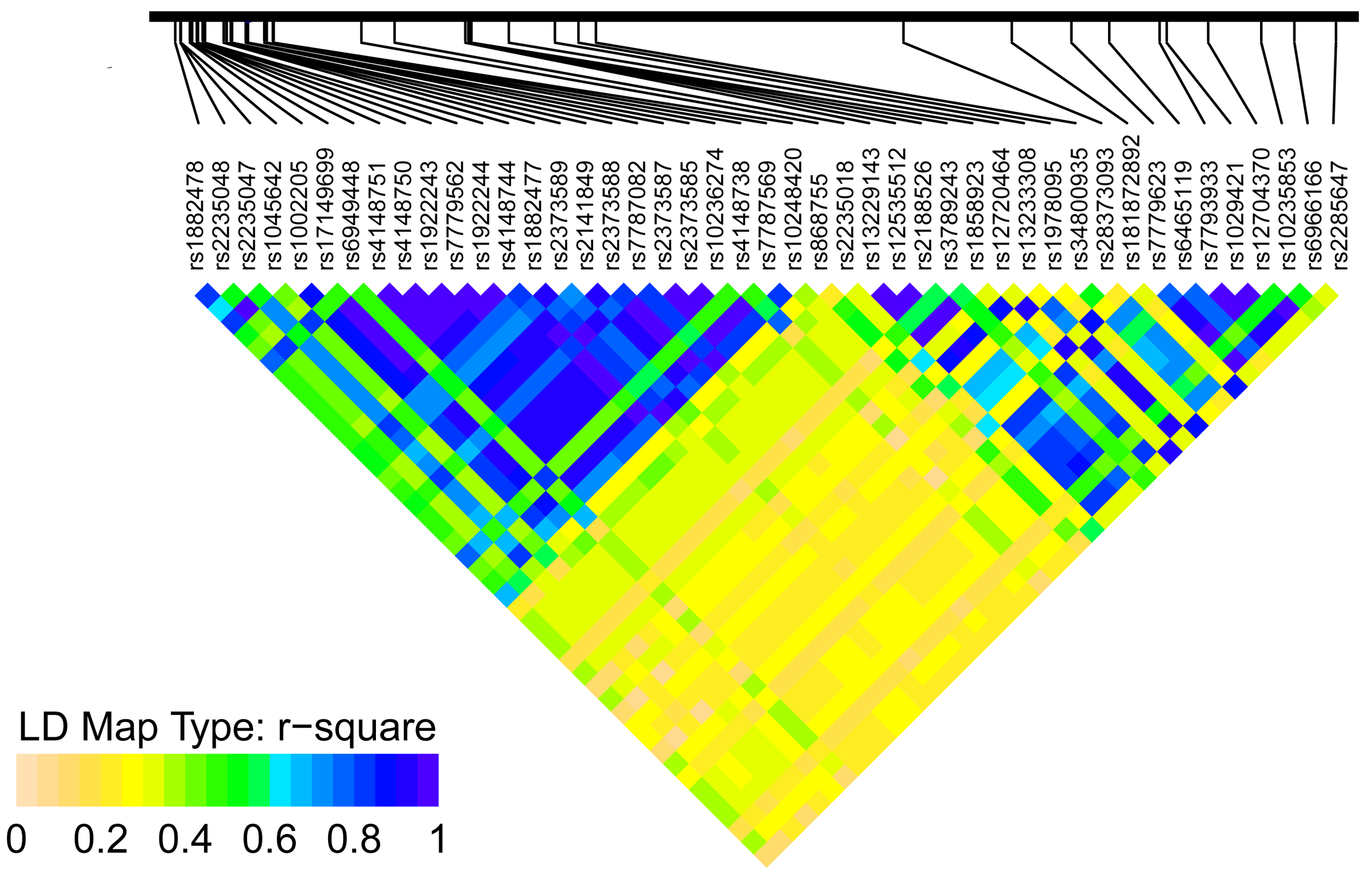

Supplement: Figure S6 — LD plot of SNPs with top-ranked BFs in combined Chinese Han (CHB and CHS) of 1000 Genome Phase I. The colors indicate the strength of pairwise LD according to r 2 metrics. (TIF) [file pone.0046295.s006.tif]
