# Supplementary material for: Gene-Wide Characterization of Common Quantitative Trait Loci for ABCB1 mRNA Expression in Normal Liver Tissues in the Chinese Population
Source: PLoS One. 2012 Sep 26;7(9):e46295. doi: 10.1371/journal.pone.0046295 (PMC3458811; doi:10.1371/journal.pone.0046295)
Supplement: Table S1 — Reference panels and detailed option-settings used in IMPUTE. (DOC) [file pone.0046295.s007.doc]

**Table S1.** Reference panels and detailed option-settings used in IMPUTE.

| **Reference Panel** | **1kG PhaseI interim**  **(Jun 2011)** | **Hapmap Phase** III **release2**  **(Feb 2009)** |
| --- | --- | --- |
|  | SNP positions in NCBI b37 coordinates | SNP positions in NCBI b36 coordinates |
|  | ASW; CEU; CHB; CHS; CLM; FIN; | ASW; CEU; CHB; CHD; GIH; JPT |
|  | GBR; IBS; JPT; TSI; YRI | LWK; MEX; MKK; TSI; YRI |
|  | Tatol: 2188 Haplotypes | Tatol: 2022 Haplotypes |
| **Basic Options** |  | |
| Interval for inference | Chr7: 86978000…87478000 | Chr7: 86830000…87330000 |
| Buffer region | 250kb (by default) | 250kb (by default) |
| Ne | 20000 (recommended) | 15000 (recommended) |
| **MCMC Options** |  | |
| Burn-in MCMC iterations | 20 | |
| Total MCMC iterations | 30 | |
| HMM states for phasing | 100 | |
