# Supplementary material for: Gene-Wide Characterization of Common Quantitative Trait Loci for ABCB1 mRNA Expression in Normal Liver Tissues in the Chinese Population
Source: PLoS One. 2012 Sep 26;7(9):e46295. doi: 10.1371/journal.pone.0046295 (PMC3458811; doi:10.1371/journal.pone.0046295)
Supplement: Table S2 — Genotyping results of all Tag SNPs. (DOC) [file pone.0046295.s008.doc]

**Table S2.** Genotyping results of all Tag SNPs.

| **SNP** | **Position**^a^ | **A1** | **A2** | **MAF** | **P_HWE**^b^ |
| --- | --- | --- | --- | --- | --- |
| **rs4148809** | 86941199 | A | G | 0.4384 | 0.6396 |
| **rs2888611** | 86941606 | G | C | 0.2466 | 0.2061 |
| **rs12673662** | 86948287 | G | C | 0.1712 | 1 |
| **rs45565442** | 86948434 | G | A | *0.03425* | 1 |
| **rs6946119** | 86966801 | G | A | *0.08904* | 1 |
| **rs6978925** | 86974951 | T | C | 0.1164 | 0.5838 |
| **rs1882478** | 86974954 | C | T | 0.4658 | 0.2422 |
| **rs1045642** | 86976581 | A | G | 0.411 | 0.8093 |
| **rs1922243** | 86981440 | T | C | 0.3904 | 1 |
| **rs55912869** | 86991073 | G | A | *0.0274* | 1 |
| **rs2373588** | 86991096 | T | C | 0.3904 | 1 |
| **rs2032582** | 86998554 | T | A/G | 0.4247 | 1 |
| **rs4148738** | 87000985 | C | T | 0.4315 | 1 |
| **rs12668877** | 87004940 | A | G | 0.1096 | 1 |
| **rs2091766** | 87012440 | T | C | 0.274 | 1 |
| **rs1128503** | 87017537 | G | A | 0.2877 | 0.7751 |
| **rs868755** | 87027866 | A | C | 0.4521 | 1 |
| **rs2520464** | 87039022 | C | T | *0.05479* | *2.58×10^-7^* |
| **rs11763872** | 87055151 | A | G | 0.2466 | 1 |
| **rs12535512** | 87058270 | A | G | 0.4452 | 0.343 |
| **rs3789243** | 87058822 | A | G | 0.3904 | 1 |
| **rs3213619** | 87068129 | C | T | *0.02055* | 1 |
| **rs2188524** | 87068371 | G | A | *0.06849* | 0.281 |
| **rs1978095** | 87089577 | G | A | 0.2329 | 1 |
| **rs10231033** | 87099516 | C | T | *0.06164* | 1 |
| **rs10276499** | 87099672 | G | A | *0.08904* | 1 |
| **rs10264856** | 87100517 | A | G | *0.08904* | 1 |
| **rs10233247** | 87109774 | C | T | *0.06849* | 1 |
| **rs3747802** | 87180522 | C | T | *0.0274* | 1 |
| **rs17160359** | 87184755 | A | C | *0.04795* | 1 |
| **rs2157927** | 87198864 | T | G | *0.0137* | 1 |
| **rs10275625** | 87203184 | T | C | *0.06164* | 1 |
| **rs11976031** | 87203218 | T | A | *0.06164* | 1 |
| **rs17251003** | 87208119 | C | T | 0.137 | 0.6106 |
| **rs28535601** | 87208205 | A | G | *0.06164* | 1 |

^a^ Physical positions of SNPs are based on Human Reference Genome Sequence Build 36.

^b^ *P* values of Hardy-Weiberg equilibrium test.
