# Supplementary material for: Gene-Wide Characterization of Common Quantitative Trait Loci for ABCB1 mRNA Expression in Normal Liver Tissues in the Chinese Population
Source: PLoS One. 2012 Sep 26;7(9):e46295. doi: 10.1371/journal.pone.0046295 (PMC3458811; doi:10.1371/journal.pone.0046295)
Supplement: Table S3 — Tag SNP association with ABCB1 gene expression using linear regression analysis. Physical positions of the SNPs are based on Human Reference Genome Sequence Build 36. Nominal significant P values<0.05 are shown in italics. (DOC) [file pone.0046295.s009.doc]

**Table S3.** Tag SNP association with *ABCB1* gene expression using linear regression analysis. Physical positions of the SNPs are based on Human Reference Genome Sequence Build 36. Nominal significant *P* values < 0.05 are shown in italics.

| **SNP** | **Position** | **A1** | **A2** | **MAF** | **BETA** | **R^2^** | **P_Value** | **Bonferroni** | **FDR** |
| --- | --- | --- | --- | --- | --- | --- | --- | --- | --- |
| **rs4148809** | 86941199 | A | G | 0.4384 | -0.001663 | 0.02999 | 0.1429 |  |  |
| **rs2888611** | 86941606 | G | C | 0.2466 | 0.0001288 | 0.000106 | 0.9313 |  |  |
| **rs12673662** | 86948287 | G | C | 0.1712 | 0.001453 | 0.01235 | 0.3493 |  |  |
| **rs6978925** | 86974951 | T | C | 0.1164 | 0.0005231 | 0.001021 | 0.7884 |  |  |
| **rs1882478** | 86974954 | C | T | 0.4658 | -0.003906 | 0.1338 | *0.00146* | **√** | **√** |
| **rs1045642**  **3435C>T** | 86976581 | A | G | 0.411 | -0.003389 | 0.1205 | *0.00262* | **√** | **√** |
| **rs1922243** | 86981440 | T | C | 0.3904 | 0.003997 | 0.1576 | *0.00051* | **√** | **√** |
| **rs2373588** | 86991096 | T | C | 0.3904 | 0.004146 | 0.1696 | *0.00029* | **√** | **√** |
| **rs2032582**  **2677G>A/T** | 86998554 | T | A/G | 0.4247 | -0.003314 | 0.111 | *0.00397* |  | **√** |
| **rs4148738** | 87000985 | C | T | 0.4315 | -0.003547 | 0.1246 | *0.00219* | **√** | **√** |
| **rs12668877** | 87004940 | A | G | 0.1096 | 0.001754 | 0.01276 | 0.3414 |  |  |
| **rs2091766** | 87012440 | T | C | 0.274 | -7.80E-05 | 4.88E-05 | 0.9532 |  |  |
| **rs1128503** | 87017537 | G | A | 0.2877 | 0.0002165 | 0.000373 | 0.8711 |  |  |
| **rs868755** | 87027866 | A | C | 0.4521 | -0.003141 | 0.1025 | *0.00575* |  | **√** |
| **rs11763872** | 87055151 | A | G | 0.2466 | -4.78E-05 | 1.72E-05 | 0.9722 |  |  |
| **rs12535512** | 87058270 | A | G | 0.4452 | 0.004511 | 0.1812 | *0.00017* | **√** | **√** |
| **rs3789243** | 87058822 | A | G | 0.3904 | 0.002736 | 0.07387 | *0.02002* |  | **√** |
| **rs1978095** | 87089577 | G | A | 0.2329 | 0.004805 | 0.1729 | *0.00025* | **√** | **√** |
| **rs17251003** | 87208119 | C | T | 0.137 | -0.000797 | 0.003366 | 0.6259 |  |  |
