# Supplementary material for: Gene-Wide Characterization of Common Quantitative Trait Loci for ABCB1 mRNA Expression in Normal Liver Tissues in the Chinese Population
Source: PLoS One. 2012 Sep 26;7(9):e46295. doi: 10.1371/journal.pone.0046295 (PMC3458811; doi:10.1371/journal.pone.0046295)
Supplement: Table S4 — Association results of imputed loci with top-ranked BFs using HapMap III datasets as reference panel. SNPs marked with an asterisk denote Tag SNPs. P values were produced by 100000 random permutations. Physical positions of the SNPs are based on Human Reference Genome Sequence Build 36. (DOC) [file pone.0046295.s010.doc]

**Table S4.** Association results of imputed loci with top-ranked BFs using HapMap III datasets as reference panel. SNPs marked with an asterisk denote Tag SNPs. *P* values were produced by 100000 random permutations. Physical positions of the SNPs are based on Human Reference Genome Sequence Build 36.

| **SNP** | **Position** | **A1 A2** | **Infor (Impute)** | **MAF** | **Log10(BF)** | **P Value** |
| --- | --- | --- | --- | --- | --- | --- |
| **rs17149699** | 86979687 | T C | 0.97 | 0.396 | 1.846 | 1.80E-04 |
| **rs13233308** | 87082896 | C T | 0.982 | 0.462 | 1.651 | 2.70E-04 |
| **rs10236274** | 87000277 | G A | 0.989 | 0.388 | 1.635 | 2.90E-04 |
| **rs7779623** | 87229697 | A G | 0.959 | 0.464 | 1.573 | 3.30E-04 |
| **rs1858923** | 87059152 | A G | 0.989 | 0.451 | 1.572 | 3.70E-04 |
| **rs4148750** | 86981211 | C T | 0.973 | 0.403 | 1.553 | 2.80E-04 |
| **rs12720464** | 87069789 | T C | 0.995 | 0.232 | 1.483 | 3.80E-04 |
| **rs2235047** | 86976468 | C A | 0.92 | 0.403 | 1.472 | 4.40E-04 |
| **rs7779562** | 86982752 | C G | 0.984 | 0.395 | 1.471 | 3.60E-04 |
| **rs12704370** | 87268605 | A G | 0.969 | 0.452 | 1.45 | 5.30E-04 |
| **rs2285647** | 87304907 | A G | 0.934 | 0.236 | 1.413 | 4.20E-04 |
| **rs10248420** | 87002922 | G A | 0.988 | 0.418 | 1.337 | 6.30E-04 |
| **rs1029421** | 87256797 | C T | 0.933 | 0.475 | 1.209 | 1.07E-03 |
| **rs6949448** | 86979750 | T C | 0.911 | 0.425 | 1.105 | 1.15E-03 |
